# Upper Gastrointestinal Sensitization And Symptom Generation

**DOI:** 10.25122/jml-2019-0111

**Published:** 2019

**Authors:** Alina Suciu, Stefan-Lucian Popa, Dan-Lucian Dumitrascu

**Affiliations:** Second Medical Department “Iuliu Hatieganu” University of Medicine and Pharmacy Cluj-Napoca, Romania

**Keywords:** Functional Gastrointestinal Disorders, FGID, Brain-Gut Interaction, Sensitization, Visceral Hypersensitivity, ASICs - Acid-sensing ion channels, CHS - Cannabinoid hyperemesis syndrome, CNS - Central nervous system, CNVS - Chronic nausea vomiting syndrome, CVS - Cyclic vomiting syndrome, EPS- Epigastric pain syndrome, FGID - Functional gastrointestinal disorders, GNB3 - G-protein-coupled receptor in the brain-gut axis, HPA - Hypothalamic-pituitary-adrenal, NMDA N-methyl-D-aspartate receptor, PDS - Postprandial distress syndrome, PTSD - Posttraumatic stress disorder, TRPV1 - The transient receptor potential vanilloid 1.

## Abstract

Functional gastrointestinal disorders (FGIDs) are a highly prevalent group of heterogeneous disorders, and their diagnostic criteria are symptom-based, with the absence of anatomical and biochemical abnormalities of the gastrointestinal tract. Chronic visceral symptoms are common both in patients with an identifiable organic disease but also in FGID patients. Patients suffering from upper gastrointestinal functional disorders typically present with various symptoms such as early satiety, postprandial fullness, bloating, nausea, vomiting, and epigastric pain. Considering their increasing prevalence, difficulties in diagnosis, and low quality of life, FGIDs have become an emerging problem in gastroenterology. We aimed to provide an updated summary of pathways involved in visceral sensitization. We examined the recent literature searching for evidence of the most important studies about the mechanisms underlying gastrointestinal symptom generation and sensitization.

## Introduction

The increasing burden of visceral symptoms has generated a growing interest of researchers and clinicians in studying the origins of FGID symptoms, especially visceral pain. The mechanisms involved in the perception of gastrointestinal pain and discomfort are complex. FGID patients typically present with various symptoms such as early satiety, postprandial fullness, bloating, nausea, vomiting, and epigastric pain. Considering their increasing prevalence, difficulties in diagnosis, and low quality of life, FGIDs have become an emerging problem in gastroenterology.

Some primary visceral afferent fibers have a significant efferent function, but their role in the physiology and pathophysiology of the viscera has not been widely studied [2]. The most evaluated function of visceral receptors is to convey information from the viscera to the central nervous system. Accordingly, the first conscious sensations that arise from the viscera are discomfort and pain [3]. The cell bodies of primary visceral afferent neurons are contained in the nodose ganglia (vagal afferents) and dorsal root ganglia (spinal afferents) [4].

The viscera receive a dual innervation from vagal and spinal primary afferent neurons. The central terminals of vagal sensory neurons are in the brain stem. A large part of the literature, however, suggests that most primary visceral afferents are contributing to altered sensations from the viscera in pathophysiological conditions [1-3]. The terminals (receptors) of primary visceral afferent neurons are located in the mucosa, muscularis, and serosa (mesentery) of hollow tubular organs [3]. Visceral receptors have no end organs or morphological specialization. Visceral afferent neuron terminals are placed to respond to luminal and local chemical stimuli and mechanical ones. These altered sensations are considered to represent visceral hyperalgesia [4-6].

Chronic visceral symptoms are common both in patients with an identifiable organic disease and also in those without structural or biochemical abnormalities.

In the last two decades, most experts believe that FGID with abdominal or chest pain may be a consequence of one or more mechanisms. Those mechanisms include abnormal motility, visceral hypersensitivity [7-12], microscopic inflammation [13], disorders of the brain-gut interaction [10, 14], psycho-social factors [15, 16], genetic susceptibility [17-19] and postinfectious, neuromuscular and neurotransmitter dysfunction [20-24].The Rome IV Criteria classified upper gastrointestinal functional disorders (table 1) in esophageal disorders (functional chest pain, functional heartburn, reflux hypersensitivity, globus, functional dysphagia) and gastric disorders (functional dyspepsia, belching disorders, nausea and vomiting disorders, rumination syndrome) [1].

**Table 1:** Classification of upper gastrointestinal functional disorders according to Rome IV criteria.

A. Esophageal Disorders	B. Gastroduodenal disorders
Functional chest pain	**I. Functional dyspepsia**
Functional heartburn	Postprandial distress syndrome(PDS)
Reflux hypersensitivity	Epigastric pain syndrome (EPS)
Globus	**II. Belching disorders**
Functional dysphagia	Excessive supragastric belching
	Excessive gastric belching
	**III. Nausea and vomiting disorders**
	Chronic nausea vomiting syndrome(CNVS)
	Cyclic vomiting syndrome (CVS)
	Cannabinoid hyperemesis syndrome (CHS)
	Rumination Syndrome

We examined journal entries in PubMed from 2009 to 2019, focused on sensitization, symptom generation and visceral hyperalgesia in upper gastrointestinal functional disorders, providing an updated summary of the pathways involved in visceral sensitization.

Keywords of the search were: functional disorder, gastrointestinal, FGID, Brain-Gut Axis, Sensitization, Visceral hypersensitivity. We included in this narrative review original papers, reviews and meta-analyses.

### Symptoms and mechanism of production

**Figure1:**
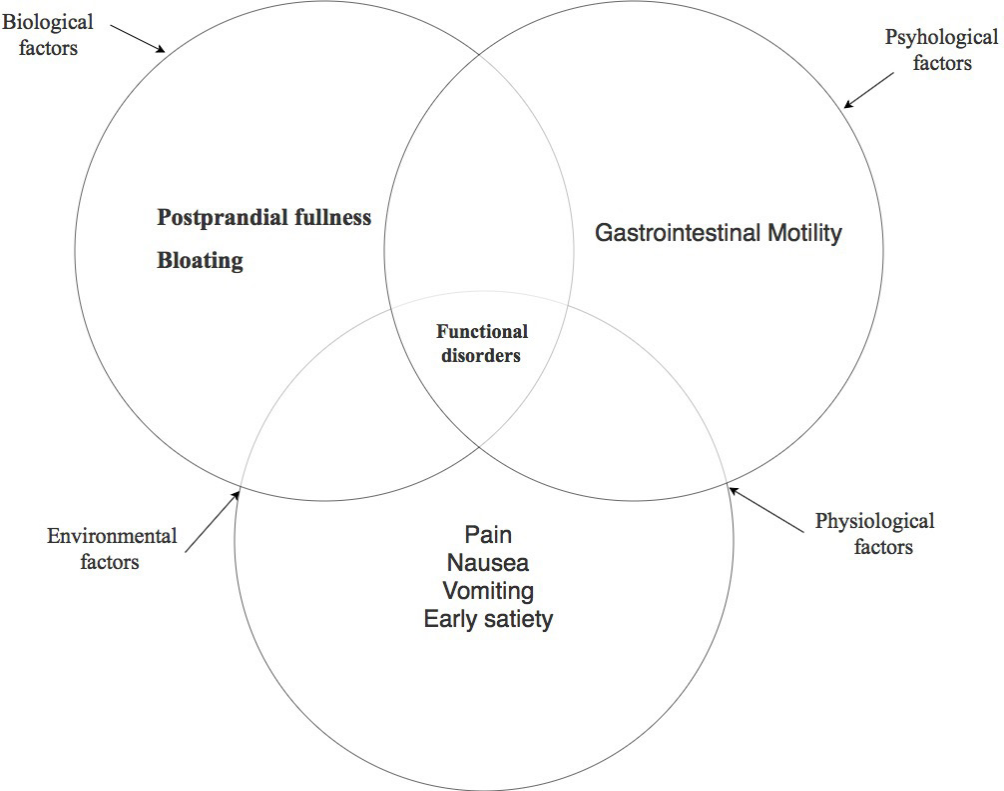
Functional gastrointestinal disorders symptomatology.

Typically, patients that are suffering from upper gastrointestinal (GI) disorders present with various symptoms such as early satiety, postprandial fullness, bloating, nausea, vomiting, and epigastric pain (Figure 1) [25]. Upper GI disorders have become an emerging problem in gastroenterology considering their increasing prevalence, difficulties in diagnosis, and patients’ low quality of life.

Individuals with functional gastrointestinal disorders report having experienced psychological trauma (e.g., sexual and/or physical abuse or assault) more often than patients with organic gastrointestinal diseases or healthy individuals [26-28].

**Figure 2:**
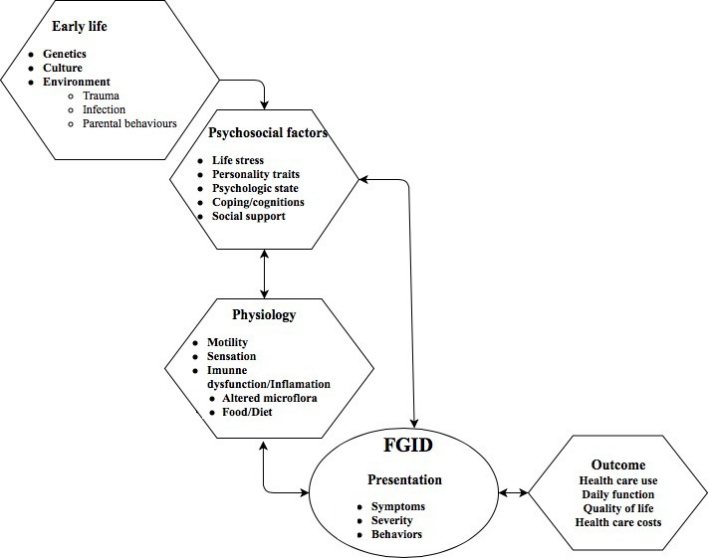
Pathogenesis of functional GI disorders.

Multiple psychosocial and biological mechanisms have incriminated for the occurrence of FGID (Figure 2) in individuals exposed to interpersonal trauma. Traumatic events have their role in symptoms expression, enhancing reliance on maladaptive coping styles, and by triggering the onset of psychiatric conditions known to impact pain sensitivity.

The most frequent example is posttraumatic stress disorder (PTSD) or major depressive disorder [29]. Individuals exposed to trauma tend to exhibit heightened autonomic and hypothalamic-pituitary-adrenal (HPA) responses to physical and psychological stressors. The experience of psychological trauma may impact risk for FGIDs by altering corticolimbic pain modulation [30], as well as visceral and central sensitization [31].

### Central GI sensitization

Some researchers observed heightened central sensitization in patients with FGIDs [32], which is characterized by increased responsiveness to painful stimuli resulting from alterations within the central nervous system (CNS) and represents one possible mechanism linking FGIDs and trauma.

Central sensitization can be indexed through a pattern of increased perceived pain intensity in response to repetitive stimulation administered at a constant intensity, termed temporal summation [33]. Based on current scientific research, some hypotheses have been proposed to explain the mechanism of visceral hypersensitivity:

• peripheral sensitization represented by sensitization of GI afferent nerves,
• central sensitization represented by sensitization of spinal cord dorsal horn neurons,
• misinterpretation of non-noxious sensation as noxious due to hypervigilance
• altered descending excitatory or inhibitory influences on the spinal cord nociceptive neurons [6].

In the periphery, inflammatory mediators activate and sensitize nociceptive afferent nerves by reducing their transduction thresholds and by inducing the expression and recruitment of hitherto silent nociceptors culminating in an increase in pain sensitivity at the site of injury known as primary hyperalgesia.

The main mechanisms underlying maladjusted sensitization include changes in peripheral neuroimmune interactions, nerve injury, peripheral inflammation, and dysfunctions of acid-sensing ion channels (ASICs), ATP-gated ion channels (P2X, P2Y) voltage-gated sodium channels (VGSC) [30-35]. Central mechanisms of sensitization are modulated by substance P, N-methyl-D-aspartate (NMDA) receptor, neurokinin B, PGE2, psychological factors, hypervigilance, and endogenous pain modulation [9, 10, 23]. Centrally, secondary hyperalgesia, defined as an increase in pain sensitivity in anatomically distinct sites, occurs at the level of the spinal dorsal horn.

Several neurotransmitters, such as N-methyl-d-aspartate [9], serotonin [22], and adenosine [23, 24], have been proposed as mediators for this symptom. Visceral hypersensitivity, either due to peripheral or central sensitization, has been postulated as a mechanism for functional gastrointestinal pain [8, 18].

### Perifepheral sensitisation

Pain and gastrointestinal discomfort are the leading symptoms in the multitude of upper GI disorders. Generally, noxious stimuli are encoded by nociceptors located in the organs. When the nociceptor receives a noxious stimulus strong enough to cause a depolarization, an action potential is generated and transmitted along the first-order neuron to the dorsal horn of the spinal cord [34]. Most visceral nociceptors are nonspecific (polymodal) and respond to different mechanical, chemical, electrical, thermal, and ischemic stimuli [35]. Nerve fibers may respond to either phasic or tonic distension of the gut, and this has, to some degree, been confirmed in animal and human studies [36-41]. Some fibers, especially the mucosal fibers, are adapting to a given stimulus and give no response when the stimulus is maintained, whereas afferents in the muscular layers generally show less adaptation [38].

Potential mechanisms of dyspepsia that have been recently described include hypersensitivity to gastric distension, hypersensitivity to small intestinal fat, gut hormones, and hypersensitivity to acid.

The transient receptor potential vanilloid 1 (TRPV1) activation can be lowered by hydrogen ions and inflammatory mediators [42]. Acid-sensitive receptors in the gut consist mainly of three groups: TRPV1 (which is temperature, as well as acid-sensitive), acid-sensing ion channels (ASICs), and purine receptors [43]. All the afferent nerves projecting to the spinal cord terminate in the dorsal horn. From here, the stimulus transmits cephalad through the spinal cord pathways and synapses to the third-order neuron in the brain or brainstem [44].

There is a close interaction between GI afferents and those from the somatic, autonomic, and enteric nervous systems. The activity in the GI organs does not usually reach the higher brain centers, except for information due to the filling of the esophagus, stomach, and rectum. When the organs are potentially in danger, for example, due to diseases, symptoms such as discomfort and pain are sensed [45]. Furthermore, mechanisms involved in the occurrence of heartburn include esophageal hypersensitivity, peripheral or central sensitization, microscopic alteration of the esophageal mucosa, and dilated intercellular spaces. From the spinal cord, pain transmits to the brain through several distinct pathways but, most afferents travel in the spinothalamic tract to the thalamus and from the thalamus project to the insula, hypothalamus, and amygdala as well as to higher cortical levels such as cingulate and prefrontal cortices [44]. The anterior cingulate cortices and prefrontal cortices are a part of the medial pain system, which mediates the affective, emotional, and cognitive components of the pain experience [46]. Peripheral nociceptor sensitization underlies the hyperalgesia that develops around an injury site. Like in the cutaneous system, upper GI afferent fibers may become sensitized by endogenous chemicals, resulting in an increase in their responsiveness to a given stimulus and/or an increase in the spontaneous activity [47].

Various inflammatory mediators, including protons, prostaglandins, serotonin, and histamine, are released in case of local inflammation. This leads to increased afferent activity to the spinal cord and exacerbation of the pain [47].

Moreover, an upregulated expression of nociceptors such as sodium channels, TRPV1, ASICs, and purine receptors are seen during inflammation. As a consequence of all of these changes, the pain sensitivity at the site of inflammation is increased [48, 49].

Enhanced spinal input can activate intracellular signaling cascades within the spinal dorsal horn neurons. This results in increased synaptic efficacy and is known as central sensitization [50]. The input leads to the activation of the N-methyl-D-aspartic acid receptor and results in changes in the resting potential of the second-order neuron [44]. Blocking the N-methyl-D-aspartic acid receptor has been shown to prevent experimentally acid-induced central sensitization [51].

## Discussion

The mechanisms involved in the perception of gastrointestinal pain and discomfort are complex. Apparently, visceral receptors have no end organs or morphological specialization.

Visceral afferent neuron terminals are placed to respond to luminal and local chemical stimuli and mechanical ones. These altered sensations are considered to represent visceral hyperalgesia.

Visceral hyperalgesia is a complex form of hypersensitivity involving complex mechanisms. Mechanisms involved in the occurrence of chest pain include gastroesophageal reflux, esophageal motility disorders, and esophageal hypersensitivity.

This phenomenon takes place between visceral organs that share their central afferent termination; that is why central sensitization plays an important role [51].

Besides changes at the spinal level, changes in the cortical processing of pain may be involved in these mechanisms [52]. Irritation of peripheral nerve trunks (neuritis) or direct damage (neuropathy) contributes with an altered input to the central nervous system. In animal models of somatic nerve mononeuropathy or neuritis, hyperalgesia is characteristically produced and is long-lasting [48-52]. Ligand and voltage-gated channels in sensory neurons may be altered subsequent to a nerve injury and thus contributes to the occurrence of pain. Candidate channels include voltage-gated sodium and calcium channels, acid-sensing and temperature-sensing ion channels, and ion channels gated by endogenous ligands such as serotonin or ATP [40-45].

Many ion channels have been cloned, opening the possibility of precise studying of molecular and pharmacological processes. A new promising research direction is represented by the genetic variation in GNB3 (G-protein-coupled receptor in the brain-gut axis) and ADRB2 (which is a mediator of the stress response) that can explain the abnormal effect and esophageal mucosal injury in patients with esophageal symptoms [53]. Current evidence for the relation between genetic, immunological, psychosocial, and infectious factors involved in the occurrence of FGID is insufficient because of the limited number of prospective studies with detailed analysis of patients and matched controls but also the limited data provided by experimental animal studies

## Conclusions

FGIDs are characterized by chronic complaints arising from disorganized brain-gut interactions leading to dysmotility and hypersensitivity. Chronic visceral symptoms are common both in patients with an identifiable organic disease but also in those without structural or biochemical abnormalities. Visceral pain hypersensitivity induced by central sensitization results from increased central neuronal excitability.

## Conflict of Interest

The authors confirm that there are no conflicts of interest.
